# Effect of tourniquet technique on postoperative delirium in elderly patients with total knee arthroplasty: a randomized single-blind controlled trial

**DOI:** 10.1186/s12871-022-01938-5

**Published:** 2022-12-20

**Authors:** Wei Ran, Shuzhen Li, Ruixue Yuan, Huan Luo, Ping Li, Jin Gao

**Affiliations:** Department of Anesthesiology, The First Affiliated Hospital of Chongqing Medical University, Chongqing, 400016 China

**Keywords:** Postoperative delirium (POD), Elderly patients, Total knee arthroplasty (TKA), Tourniquet technique, Postoperative rehabilitation

## Abstract

**Background:**

The tourniquet technique is often used in total knee arthroplasty (TKA). However, its effect on postoperative delirium (POD) in elderly patients undergoing TKA is unknown.

**Methods:**

This prospective randomized controlled trial assessed the eligibility of 245 elderly patients. A total of 197 patients who met the inclusion criteria were randomly divided into a tourniquet group (*n* = 98) and a non-tourniquet group (*n* = 99). The primary outcome was the incidence of POD within 72 h after surgery. The secondary outcome was the quality of rehabilitation, including inflammatory reaction, postoperative pain, hypoproteinemia and anemia.

**Results:**

Of 245 patients, 184 patients completed this clinical trial, with 92 cases in each group. There were 14 patients (15.22%) with POD in the tourniquet group and 5 patients (5.43%) in the non-tourniquet group (95% CI 1.076 to 9.067, *P* = 0.029). The changes in white blood cell count (WBC), the proportion of neutrophils (NEUT%), c-reactive protein (CRP), interleukin-6 (IL-6) and middle patellar circumference in the tourniquet group were higher than those in the non-tourniquet group (*P* < 0.05). The visual analog scale (VAS) at rest and activity in the tourniquet group were higher than those in the non-tourniquet group (*F* = 170.102, *P* < 0.001 *F* = 75.391, *P* < 0.001). There were 41 (44.57%) patients with hypoproteinemia in the tourniquet group and 26 (28.26%) in the non-tourniquet group (95% CI 1.106 to 3.765, *P* = 0.022).

**Conclusion:**

The application of the tourniquet technique in elderly patients with TKA procedures increased the incidence of POD. This may be attributed to the increased inflammatory reaction, severe postoperative pain and hypoproteinemia caused by the tourniquet technique.

**Trial registration:**

Clinical trial registration number: ChiCTR2100045711.

Full date of the first registration: 23/04/2021.

## Introduction

Postoperative delirium (POD) is a common postoperative complication that is characterized by fluctuations in consciousness, cognitive decline, impaired memory, disorientation, and sleep–wake cycle disorder [1–3]. The incidence of POD has been reported to be approximately 10–25%, especially after orthopedic and cardiac surgery [4]. Although most patients have a short course of illness, it can have devastating consequences, such as decreased quality of life, increased long-term risk of Alzheimer's disease [5, 6], and accelerated cognitive decline [7]. Therefore, it is important to identify the risk factors for POD.

Previous studies have shown a high incidence of POD in elderly patients (≥ 65 years old) with hip fracture and cardiac surgery [8–10], with major risk factors including advanced age, length of nursing home residence, prior cognitive impairment, psychiatric disorders, cerebrovascular disease, end-stage renal failure, low albumin (ALB), high American Society of Anesthesiologists (ASA) classification, and intraoperative blood transfusion [11, 12].

Total knee arthroplasty (TKA) also has a high incidence of POD [13, 14]. Using a tourniquet technique may reduce bleeding during the TKA procedure, but the compression of the tourniquet on the artery and vein of the thigh may increase knee swelling and postoperative pain and increase the risk of thrombosis [15, 16]. These changes may lead to more complications after surgery. In our daily clinical work, we casually observed different rates of POD between the TKA tourniquet and non-tourniquet groups. Therefore, we hypothesized that the use of a tourniquet technique during TKA might affect the incidence of POD. Therefore, the purpose of this study was to investigate whether the tourniquet technique affects the incidence of POD in elderly patients undergoing TKA.

## Materials and methods

### Study design and participants

This prospective randomized controlled trial assessed the eligibility of 245 elderly patients. A total of 197 patients who met the inclusion criteria were randomly divided into a tourniquet group and a non-tourniquet group, and the study was conducted in the First Affiliated Hospital of Chongqing Medical University from May 2021 to February 2022. Written informed consent was obtained from all patients prior to the study, which was conducted in accordance with the Declaration of Helsinki.

#### Inclusion criteria

Patients undergoing TKA with general anesthesia; aged ≥ 65 years old; 18 ≤ body mass index (BMI) ≤ 40 kg/m^2^; ASA classification I-III; and no history of drug allergy.

#### Exclusion criteria

History of mental illness; difficulty communicating and understanding the relevant scales; history of allergy to drugs used in this trial; severe complications or inability to cooperate during or after surgery.

### Randomization and blinding

During the surgical consultation, each patient underwent an inclusion/exclusion interview and was able to proceed if all inclusion criteria and no exclusion criteria were met. Written informed consent was provided to the surgeon at that time. A 1:1 randomized design was used to assign patients to the experimental and control groups.

All patients were sequentially numbered 1–245, and patients with odd numbers were included in the experimental group and those with even numbers were placed in the control group. The tourniquet technique was used in the experimental group, while the non-tourniquet technique was used in the control group.

The TKA procedure was performed by the same team of 4 surgeons. The patients, data collector and care team were blinded to group assignment, while the anesthesiologists and surgeons were unblinded participants who were not otherwise involved in the study.

### Perioperative management

All patients were educated about the outcome scales one day before the surgery, including the Mini-mental State Examination (MMSE), visual analog scale (VAS) and confusion assessment method for the ICU (CAM-ICU) scale. The pre-surgery MMSE score and patellar circumference data were collected at this time.

All patients received general anesthesia with endotracheal intubation. The monitoring techniques used by all patients included electrocardiogram (ECG), pulse oxygen saturation (SpO_2_), body temperature (T), invasive arterial blood pressure (IBP) and the Narcotrend index.

#### Anesthesia management

Induction: midazolam 0.04 mg/kg, sufentanil 0.5 µg/kg, propofol 1.5 mg/kg, and rocuronium 0.6 mg/kg. Anesthesia was maintained by total intravenous anesthesia (propofol 4–6 mg/kg∙h, remifentanil 0.2–1 µg/kg∙min), while sufentanil (5–10 µg) and rocuronium (10–20 mg) were intermittently injected to maintain the Narcotrend phase at D2-E1 (recommended depth of anesthesia maintenance). Before skin suture, the patients were given parecoxib sodium 40 mg as auxiliary analgesia, and tropisetron 2 mg was administered 30 min before the end of the operation to prevent vomiting. Anesthesia maintenance was stopped after incision suturing. All patients were monitored in the postanesthesia care unit for at least 1 h and then returned to the general ward.

#### Analgesia management

All patients received the same continuous femoral nerve block and cocktail analgesia. Nerve blocks were performed preoperatively by a certified regional anesthesiologist with 0.2% ropivacaine (20 ml) under ultrasound guidance. A patient-controlled continuous nerve block pump was used for postoperative analgesia (total of 0.17% ropivacaine 300 ml, load dose 5 ml, continuous dose 5 ml/h, compression volume 5 ml, interval 45 min); if the VAS ≥ 4 and the pain was not relieved after 5 min of a bolus dose, tramadol 50 mg was immediately injected intravenously.

### Data collection

Data from the patients were collected prospectively in real time on a specific case report form by research technicians. Data collectors who were in charge of routine care were informed of the ongoing study but were blinded to the research data.

### Clinical evaluation criteria

POD: CAM-ICU score ≥ 20; anemia: male below 120 g/L, female below 110 g/L; severe postoperative pain: VAS ≥ 4; hypoproteinemia: concentration of ALB < 35 g/L. Postoperative knee swelling: Compared with the preoperative baseline, the circumference of the middle patella increased by more than 3 cm.

#### Main outcome measurements

POD was defined as delirium that occurred in the general wards during postoperative days 1 to 3, and it was assessed with the CAM-ICU twice daily (8:00–10:00 am, 18:00–20:00 pm). The incidence of POD within 72 h after surgery was calculated in the two groups.

#### Secondary outcome measurements

The quality of rehabilitation, including inflammatory reaction (white blood cell count [WBC], proportion of neutrophils [NEUT%], erythrocyte sedimentation rate [ESR], c-reactive protein [CRP], interleukin-6 [IL-6] and knee swelling), postoperative pain (VAS at rest and activity, postoperative opioid consumption, postoperative nausea and vomiting), hypoproteinemia and anemia (concentration of ALB and hemoglobin), was collected within 72 h after surgery.

### Sample size calculation

According to the preliminary results of 20 cases (10 cases in each group), we found that the incidence of POD was approximately 20% in the tourniquet group and 4% in the non-tourniquet group. To have a significance level of 5% and a power of 90%, the minimum sample size to detect a difference in the incidence of POD between the two groups was 85 cases in each group. Considering that the estimated dropout rate was 20%, we included a total of 245 patients. The sample size calculation was performed with PASS 15.0 (Stata Corp. LP, College Station, Texas, USA).

### Statistical analysis

Normally distributed variables are presented as the mean ± standard deviation (SD) and were analyzed by the Student’s t-test. Nonnormally distributed variables are presented as the median and quartile interval and were analyzed by the Mann‒Whitney U test. Categorical variables were analyzed by χ2 or Fisher's exact probability tests. Continuous measures (VAS, CAM-ICU score, ALB, hemoglobin, WBC, NEUT%, ESR, CRP, IL-6) were analyzed by repeated-measures analysis of variance (ANOVA). Patients with POD, anemia, severe postoperative pain, oral tramadol, nausea and vomiting, and knee swelling were counted, and the data were analyzed by χ2 test or Fisher's exact probability test.

All analyses used only complete cases without imputation of missing data and were performed with SPSS 22.0 (SPSS, Chicago, Illinois, USA). Pictures were drawn by GraphPad Prism 8.3.0 (San Diego, California, USA). A P value of less than 0.05 was considered significant.

## Results

**Fig. 1:**
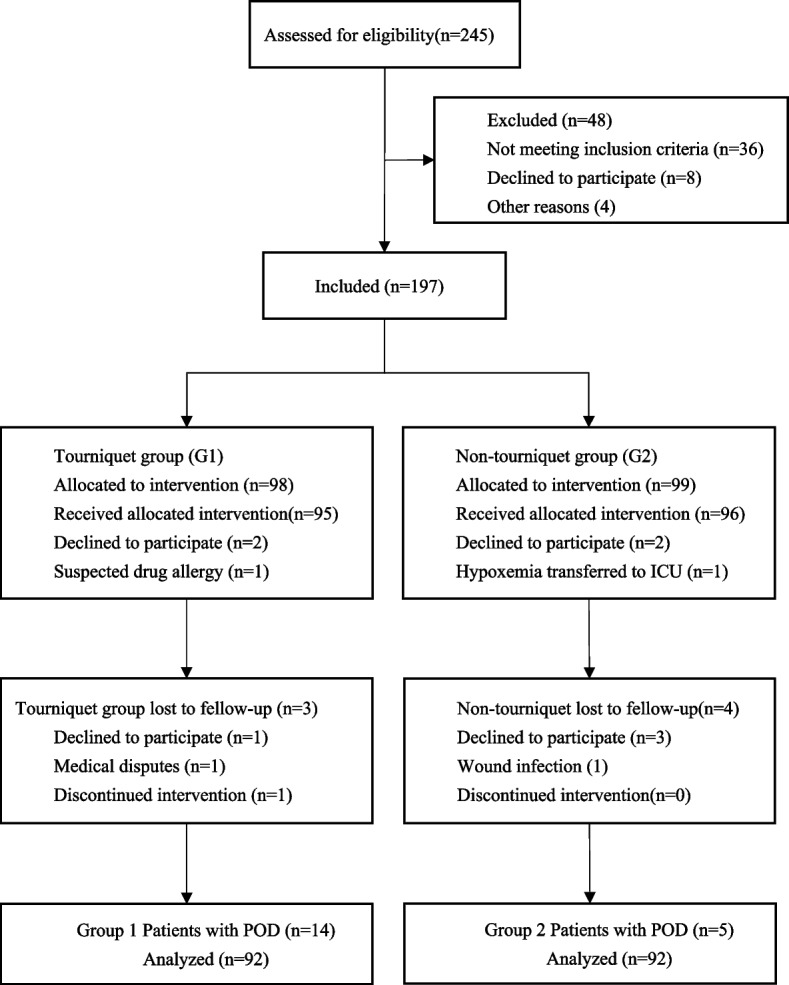
Flow diagram of the study. G1 indicates the tourniquet group, and G2 indicates the non-tourniquet group

A total of 245 patients were included in this study; 197 patients completed the group assignment, and 13 patients were excluded for various reasons. Finally, 184 patients completed the study. We summarized the demographic characteristics and surgical and anesthetic characteristics of the two groups and constructed a Consolidated Standards of Reporting Trials (CONSORT) flow diagram (Fig. 1).

### General information

There were no differences in general information, preoperative complications, educational level, baseline laboratory values or intraoperative information between the two groups, but the intraoperative blood loss in the tourniquet group was less than that in the non-tourniquet group (*P* < 0.001) (Table 1).

**Table 1:** Summary of patient characteristics

	**G1 (*n* = 92)**	**G2 (*n* = 92)**	***P* value**
Age (y)	70.88 ± 5.244	70.86 ± 5.068	0.977
Gender (male/female)	23/69	20/72	0.601
BMI (kg/m^2^)	25.37 ± 3.555	25.21 ± 3.768	0.771
Preoperative MMSE score	28.25 ± 0.567	28.11 ± 0.564	0.092
ASA Classification (II/III)	40/52	42/50	0.767
Preoperative oral opioids	10(10.9%)	13(14.1%)	0.504
Comorbidities			
Operation history (n, %)	55(59.8%)	46(50%)	0.182
Smoking history (n, %)	12(13.0%)	10(10.9%)	0.650
Drinking history (n, %)	13(14.1%)	10(10.9%)	0.504
Hypertension history (n, %)	33(35.9%)	26(28.3%)	0.269
Diabetes history (n, %)	10(10.9%)	15(16.3%)	0.282
Cerebral infarction history (n, %)	5(5.4%)	6(6.5%)	0.756
Educational level			0.894
Primary school and below (n, %)	71(77.2)	70(76.1)	
Junior middle school (n, %)	15(16.3)	17(18.5)	
Senior high school and above (n, %)	6(6.5)	5(5.4)	
Baseline laboratory values			
Preoperative hemoglobin (g/L)	122.13 ± 15.86	124.20 ± 12.96	0.335
Preoperative albumin (g/L)	40.51 ± 3.13	40.92 ± 3.10	0.370
Preoperative white blood cell (10^9^/L)	5.41[4.49 to 6.80]	5.23[4.44 to 6.07]	0.145
Preoperative ESR (mm/h)	14.29 ± 4.61	15.07 ± 3.97	0.225
Preoperative CRP (mg/L)	3.15[2.75 to 3.42]	3.06[2.43 to 3.47]	0.159
Preoperative IL-6 (ng/L)	2.64 ± 0.73	2.69 ± 0.82	0.658
Preoperative NEUT (%)	59.52 ± 8.71	57.12 ± 9.83	0.081
Intraoperative informations			
Duration of operation (min)	84.04 ± 14.66	82.58 ± 18.18	0.547
Duration of anesthesia (min)	122.13 ± 17.05	120.01 ± 20.12	0.442
Propofol dosage (mg)	344.24 ± 95.44	324.89 ± 80.98	0.140
Sufentanil dosage (mg)	40.22 ± 5.64	39.02 ± 6.43	0.182
Remifentanil dosage (mg)	863.6 ± 247.6	839.7 ± 228.9	0.497
Bleeding volume (ml)	50 [35 to 55]	80 [70 to 100]	< 0.001^*^
Infusion volume (ml)	1200 [1100 to 1600]	1200 [1100 to 1300]	0.078

Categorical variables are reported as frequencies (%) and were analyzed by the χ2 test. Normally distributed variables are reported as the mean ± standard deviation and were analyzed by independent Student’s t-tests. Nonnormally distributed variables are reported as the median [interquartile range] and were analyzed by the Mann‒Whitney U test. ^*^P < 0.05. G1 means the tourniquet group, G2 means the non-tourniquet group, BMI means body mass index, MMSE means mini-mental state examination, ESR means erythrocyte sedimentation rate, CRP means c-reactive protein, NEUT (%) means proportion of neutrophils

### Primary outcome

**Fig. 2:**
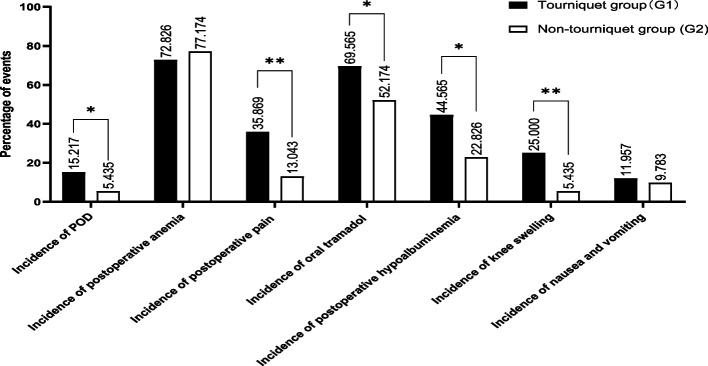
Summary of important reference indicators. Incidence of important events; all data were analyzed by the chi-square test, ^*^P < 0.05, ^**^P < 0.01

The CAM-ICU score was reduced in the non-tourniquet group on the second day after surgery (*F* = 4.267, *P* = 0.040), but there were no differences in the CAM-ICU score between the two groups on the first and third days after surgery (*F* = 0.056, *P* = 0.813 and *F* = 0.810, *P* = 0.369, respectively) (Table 2). The incidence of POD within 72 h was increased in the tourniquet group (14, 15.22% vs. 5, 5.43%, *P* = 0.029) (Table 2, Fig. 2).

**Table 2:** Summary of CAM-ICU score measurements and the incidence of POD

		**G1 (*n* = 92)**	**G2 (*n* = 92)**	**95% CI**	***F* value**	***P* value**
CAM-ICU score	T1	15.02 ± 2.94	14.85 ± 6.39	-1.272 to 1.620	0.056	0.813
	T2	14.24 ± 1.67	13.82 ± 1.05	0.019 to 0.829	4.267	0.040^*^
	T3	14.11 ± 1.71	13.90 ± 1.38	-0.245 to 0.658	0.813	0.368
	F	1.729	2.091			
	P	0.180	0.127			
Incidence of POD		14 (15.22%)	5 (5.43%)		χ^2^ = 4.754	0.029^*^
	0 ~ 36 h (n%)	10 (10.87%)	2 (2.17%)			
	36 ~ 72 h (n%)	4 (4.35%)	3 (3.26%)			

Observation points were labeled T1, T2, and T3 (T1-T3 indicate postoperative days 1–3). Continuous measurement data were analyzed by repeated measures ANOVA, ^*^P < 0.05. G1 indicates the tourniquet group, and G2 indicates the non-tourniquet group

### Secondary outcomes

#### Postoperative inflammatory reaction

The changes in WBC, NEUT%, CRP, IL-6 and middle patellar circumference in the tourniquet group were higher than those in the non-tourniquet group (*F* = 5.136, *P* = 0.025; *F* = 17.213, *P* < 0.001; *F* = 9.055, *P* = 0.003; *F* = 552.793, *P* < 0.001; and *F* = 153.839, *P* < 0.001, respectively). There were interactive effects on the WBC, CRP, IL-6 and middle patellar circumference between grouping and time (*F* = 8.572, *P* < 0.001; *F* = 5.761, *P* = 0.004; *F* = 305.466, *P* < 0.001; and *F* = 6.578, *P* = 0.002, respectively). With the extension of time, changes in the WBC, CRP, IL-6 and the circumference of the middle patella were increased in the tourniquet group. There was no difference in the ESR between the two groups, and there were no interactions on NEUT% or ESR between grouping and time (Table 3). There were 23 (25.00%) patients with knee swelling in the tourniquet group and 5 (5.43%) in the non-tourniquet group (95% CI 2.097 to 16.043, *P* < 0.001) (Fig. 2).

**Table 3:** Summary of postoperative inflammatory reaction measurements

		**G1 (*n* = 92)**	**G2 (*n* = 92)**	**95% CI**	***F* value**	***P* value**
WBC	T0	5.85 ± 1.85	5.50 ± 1.38	-0.123 to 0.828	2.140	0.145
	T1	11.94 ± 3.10	11.93 ± 2.93	-0.864 to 0.889	0.001	0.977
	T3	8.46 ± 2.55	6.99 ± 2.01	-2.144 to -0.809	19.052	< 0.001^*^
	F	253.338	253.257			
	P	< 0.001^*^	< 0.001^*^			
NEUT%	T0	59.52 ± 8.71	57.12 ± 9.83	-0.300 to 5.103	3.075	0.081
	T1	86.86 ± 6.41	85.19 ± 4.39	0.074 to 3.270	4.260	0.040^*^
	T3	72.95 ± 6.91	67.72 ± 7.93	3.602 to 7.388	22.733	< 0.001^*^
	F	326.339	375.176			
	P	< 0.001^*^	< 0.001^*^			
ESR	T0	14.29 ± 4.61	15.07 ± 3.97	-2.022 to 0.479	1.483	0.225
	T1	23.72 ± 8.00	23.74 ± 8.90	-2.484 to 2.440	0.001	0.986
	T3	36.50 ± 8.06	38.55 ± 10.58	-4.789 to 0.681	2.197	0.140
	F	243.729	268.567			
	P	< 0.001^*^	< 0.001^*^			
CRP	T0	3.09 ± 0.52	2.96 ± 0.75	-0.053 to 0.322	2.004	0.159
	T1	23.26 ± 6.79	23.50 ± 3.50	-1.821 to 1.325	0.097	0.756
	T3	47.28 ± 9.42	42.77 ± 8.60	1.960 to 7.059	12.181	0.001^*^
	F	1952.558	1741.744			
	P	< 0.001^*^	< 0.001^*^			
IL-6	T0	2.64 ± 0.73	2.69 ± 0.82	-0.277 to 0.175	0.197	0.658
	T1	23.36 ± 5.29	11.94 ± 2.67	10.204 to 12.642	341.893	< 0.001^*^
	T3	13.86 ± 3.80	3.86 ± 1.10	9.188 to 10.814	588.920	< 0.001^*^
	F	1205.699	249.260			
	P	< 0.001^*^	< 0.001^*^			
Hemoglobin	T0	122.13 ± 15.86	124.20 ± 12.96	-6.279 to 2.148	0.935	0.335
	T1	115.11 ± 13.08	109.39 ± 13.07	1.914 to 9.521	8.797	0.003^*^
	T3	104.60 ± 13.14	99.64 ± 13.26	1.116 to 8.797	6.485	0.012^*^
	F	82.403	145.485			
	P	< 0.001^*^	< 0.001^*^			
Albumin	T0	40.51 ± 3.13	40.92 ± 3.10	-1.319 to 0.493	0.809	0.370
	T1	37.32 ± 3.17	36.35 ± 2.95	0.078 to 1.857	4.601	0.033^*^
	T3	34.61 ± 3.03	36.16 ± 3.34	-2.481 to -0.627	10.942	0.001^*^
	F	89.245	100.080			
	P	< 0.001^*^	< 0.001^*^			
Increased circumference of middle patella	T1	2.86 ± 0.24	2.56 ± 0.20	0.242 to 0.369	90.366	< 0.001^*^
	T2	3.07 ± 0.21	2.86 ± 0.15	0.156 to 0.263	60.042	< 0.001^*^
	T3	3.20 ± 0.20	2.93 ± 0.13	0.224 to 0.324	114.472	< 0.001^*^
	F	90.842	138.434			
	P	< 0.001^*^	< 0.001^*^			

Observation points were labeled T0, T1, T2, and T3 (T0 means the day before surgery, T1-T3 means postoperative days 1–3). Continuous measurement data were analyzed by repeated measures ANOVA, ^*^P < 0.05. WBC means white blood cell count, NEUT (%) means proportion of neutrophils, ESR means erythrocyte sedimentation rate, CRP means c-reactive protein

#### Postoperative pain

The VAS at rest and activity in the tourniquet group were higher than those in the non-tourniquet group (*F* = 170.102, *P* < 0.001 and *F* = 75.391, *P* < 0.001, respectively). There were interactive effects on the VAS of resting activity between grouping and time (*F* = 61.199, *P* < 0.001 and *F* = 57.405, *P* < 0.001, respectively). With the extension of time, the VAS at rest and activity decreased in the tourniquet group (Table 4). Ultimately, 33 (35.87%) patients experienced severe postoperative pain (VAS ≥ 4) in the tourniquet group, and 12 (13.04%) patients experienced severe postoperative pain in the non-tourniquet group (*P* < 0.001). Sixty-four (66.57%) patients took opioids orally in the tourniquet group, and 48 (52.17%) patients took opioids orally in the non-tourniquet group (*P* = 0.016). However, there were no differences in postoperative nausea and vomiting between the two groups (11.96% vs. 9.78%) (Fig. 2, Table 4).

**Table 4:** Summary of postoperative pain measurements

		**G1 (*n* = 92)**	**G2 (*n* = 92)**	**95% CI**	***F* value**	***P* value**
Resting VAS	T1	4.43 ± 0.52	3.38 ± 0.69	0.876 to 1.233	136.276	< 0.001^*^
	T2	3.55 ± 0.50	2.91 ± 0.46	0.501 to 0.781	81.916	< 0.001^*^
	T3	2.02 ± 0.30	1.97 ± 0.18	-0.017 to 0.125	2.277	0.133
	F	743.027	263.160			
	P	< 0.001^*^	< 0.001^*^			
Activity VAS	T1	4.68 ± 0.73	3.43 ± 0.84	1.021 to 1.479	116.311	< 0.001^*^
	T2	3.72 ± 0.64	2.95 ± 0.56	0.597 to 0.946	76.265	< 0.001^*^
	T3	2.18 ± 0.55	1.99 ± 0.23	0.072 to 0.320	9.765	0.002^*^
	F	683.439	239.956			
	P	< 0.001^*^	< 0.001^*^			
Severe pain		33 (35.87%)	12 (13.04%)		χ^2^ = 12.973	< 0.001^*^
	0 ~ 36 h (n%)	8 (8.70%)	7 (7.61%)			
	36 ~ 72 h (n%)	25 (27.17%)	5 (5.43%)			
Remedial analgesia		64 (69.57%)	48 (52.17%)		χ^2^ = 5.841	0.016^*^
	0 ~ 36 h (n%)	25 (27.17%)	22 (23.91%)			
	36 ~ 72 h (n%)	39 (42.39%)	26 (28.26%)			

Observation points were labeled T1, T2, and T3 (T1-T3 indicate postoperative days 1–3). Continuous measurement data were analyzed by repeated measures ANOVA, ^*^P < 0.05. VAS means visual analog scale

#### Postoperative hypoproteinemia and anemia

There were no differences in the concentrations of ALB and hemoglobin between the two groups before surgery (*F* = 1.135, *P* = 0.288 and *F* = 2.695, *P* = 0.102, respectively). There was an interactive effect on the concentration of ALB and hemoglobin between grouping and time (*F* = 10.014, *P* < 0.001 and *F* = 11.459, *P* < 0.001, respectively). With the extension of time, the concentration of ALB was decreased in the tourniquet group, while the concentration of hemoglobin was decreased in the non-tourniquet group (Table 3). There were 41 (44.57%) patients with hypoproteinemia in the tourniquet group and 26 (28.26%) patients in the non-tourniquet group (95% CI 1.106 to 3.765, *P* = 0.022). However, there was no difference in postoperative anemia between the two groups (72.83% vs. 77.17%) (Fig. 2).

## Discussion

Our study found that the incidence of POD was increased in the tourniquet group (15.22% vs. 5.44%, *P* = 0.029), as shown in Fig. 2. The incidence of POD in the elderly has been reported to range from 5.2% to 52.2% [4, 17]. Our study was in accordance with this finding. Previous studies have shown that intraoperative EEG monitoring can reduce the dosage of narcotic drugs, avoid deep anesthesia, and help prevent the occurrence of POD [18, 19]. A recent study that randomized patients undergoing hip replacement surgery to either regional or general anesthesia did not show any difference in POD incidence between the groups. Hence, there is evidence that general anesthesia is not a significant driver behind POD [20]. To eliminate the interference of general anesthesia on the incidence of POD, all patients in our study still underwent EEG monitoring during surgery and were maintained in the same phase D2-E1. All anesthetic doses were given by ideal body weight.

At present, the pathophysiological mechanism of POD is still unclear, and a large number of studies have shown that the neuroinflammatory response plays a key role in the occurrence of POD [21, 22]. The postoperative inflammation indices (WBC, NEUT%, CRP, IL-6, changes in patella circumference) in the tourniquet group were higher than those in the non-tourniquet group, and the incidence of POD was also higher than that in the non-tourniquet group. This may be related to the local metabolic acidosis caused by tourniquets and the large number of inflammatory mediators produced by ischemia‒reperfusion. Liu et al. [23] found that POD patients had significantly increased CRP and IL-6. Ganjifard M et al. [24] found that the inflammatory response was increased after the use of tourniquets. Our results are consistent with previous studies, suggesting that an early systemic inflammatory response may be the key to the occurrence of POD. Therefore, the increased incidence of POD after tourniquet use may be related to aggravation of the systemic inflammatory response caused by the tourniquet technique.

Some studies have also suggested that severe postoperative pain (VAS ≥ 4) is a risk factor for POD [25]. Studies in rats have shown that effective postoperative analgesia can reduce the overproduction of inflammatory cytokines in the central nervous system; thus, severe postoperative pain may increase the incidence of POD by increasing the overproduction of inflammatory cytokines in the central nervous system [26]. Previous studies have also shown that the use of tourniquets can cause muscle damage in the ischemic area, leading to severe pain and swelling [27, 28]. Our study found that there were more patients with knee swelling in the tourniquet group (23 patients or 25.00%) than in the non-tourniquet group (5 patients or 5.43%) (*P* < 0.001). We also found that VAS scores during rest and activity were significantly higher in the tourniquet group than in the non-tourniquet group (*P* < 0.001). The number of patients with severe postoperative pain requiring oral opioids in the tourniquet group was higher than that in the non-tourniquet group (*P* < 0.05). However, tramadol may lead to delirium when used in excess. In our study, there were 10 patients with POD in the tourniquet group and 2 patients in the non-tourniquet group within 36 h, while there were 4 patients with POD in the tourniquet group and 3 patients in the non-tourniquet group 36 h-72 h after surgery. There were 25 patients who took opioids orally in the tourniquet group and 22 in the non-tourniquet group within 36 h, while 39 patients took opioids orally in the tourniquet group and 26 in the non-tourniquet group 36–72 h after surgery. However, there were no significant differences in intraoperative opioid use between the two groups. Therefore, most patients with delirium experience delirium before pain treatment, and the use of low-dose tramadol does not seem to affect the incidence of POD. Thus, the use of tourniquets may increase postoperative pain and knee swelling, which may be an important reason for the high incidence of POD.

Previous studies have reported that after total hip or knee replacement in elderly patients [29], a reduction in postoperative ALB concentration can independently predict the occurrence of POD. Oh et al. [30] showed that a low concentration of ALB is considered a major risk factor for POD in surgical patients. In our study, the concentration of ALB in the tourniquet group declined, and the incidence of POD increased, which is consistent with the conclusions of previous studies. Some studies have pointed out that not using a tourniquet can increase intraoperative bleeding, and anemia will affect cerebral oxygenation, which leads to a high incidence of POD [31, 32]. However, other studies have shown that using a tourniquet can minimize intraoperative blood loss and total blood loss but will increase postoperative total blood loss [33]. Schnetler et al. [34] suggested that blood loss caused by tourniquet use during TKA may be the result of increased hidden blood loss. Another reason may be hidden blood loss after tourniquet release [35]. Our data showed that although intraoperative blood loss and the concentration of ALB was reduced in the tourniquet group, there was no difference in the incidence of anemia between the two groups. Therefore, these data indicated that tourniquet use leads to increased invisible blood loss and hypoproteinemia, leading to the high incidence of POD in the tourniquet group.

## Limitations

First, there were only a few inflammatory factors measured in this study, omitting inflammatory factors related to the central nervous system, such as IL-1β and TNFα. Second, we only assessed cognitive function after the TKA procedure in patients over 65 years old and did not include adults or other orthopedic procedures that also required tourniquets. Third, the pressure and duration of tourniquet application during surgical procedures were not recorded in detail in the database, and we were thus unable to evaluate the impact of different tourniquet pressures and duration times on POD. Some previous studies have investigated the efficacy and safety of different tourniquet pressures [36, 37] and durations [38, 39] in TKA. Fourth, this was a single-blind clinical trial, and although we tried to keep the surgical procedures and anesthesia management consistent, it could still lead to outcome bias. Fifth, a low dose of tramadol was used for remedial analgesia and midazolam for induction in this study. Although flumazenil was used to antagonize midazolam, these medications may still affect the incidence of POD. Finally, we only observed the patients for a short time and could not evaluate the long-term prognosis.

## Conclusions

In summary, the use of the tourniquet technique can increase the incidence of POD in elderly patients undergoing TKA. The increased incidence of POD may be associated with an increased inflammatory response, postoperative pain and hypoproteinemia induced by the tourniquet technique. We surmise that the disadvantages and risks associated with tourniquet use may outweigh its benefits.

## Data Availability

The datasets generated and/or analyzed during the current study are not publicly accessible but are available from the corresponding author upon reasonable request.
